# Platelet-Rich Fibrin in Fat Grafts for Facial Lipofilling: A Randomized, Controlled Split-Face Clinical Trial

**DOI:** 10.3389/fsurg.2022.793439

**Published:** 2022-04-13

**Authors:** Zhao-Xiang Zhang, Li-Hong Qiu, Nian Shi, Shao-Heng Xiong, Xian-Jie Ma, Cheng-Gang Yi

**Affiliations:** Department of Plastic Surgery, Xijing Hospital, Fourth Military Medical University, Xi'an, China

**Keywords:** platelet-rich fibrin, fat grafts, facial lipofilling, clinical trial, plastic surgery

## Abstract

**Objective:**

Previous studies have reported that platelet-rich fibrin (PRF) may enhance the efficacy of fat grafts in facial lipofilling. However, these studies either lacked objective data or were not randomized, controlled trials. Thus, we aimed to objectively evaluate the efficacy of PRF in facial lipofilling.

**Methods:**

A controlled, split-face, randomized trial (January 2018 to May 2019) based on 18 patients who underwent fat grafts for bilateral temple lipofilling was performed. Each patient received a combination of an autologous fat graft and PRF on one side and a fat graft combined with an equal volume of saline on the other side. The effects of PRF were evaluated by comparing the remaining bilateral fat graft volumes through a digital three-dimensional reconstruction technique. Improvements in the appearance and recovery time of each temple were assessed by both a surgeon and patients who were blinded to the treatment assignment. Complications were also recorded.

**Results:**

Bilateral temple lipofilling showed no evidence of fat embolism, vascular/nerve injury, infection, massive edema, or prolonged bruising. Three-dimensional reconstruction data and the assessments from both the surgeon and patients revealed no significant differences in fat graft retention volume between the PRF-positive and PRF-negative lipofilling groups. However, recovery time in the PRF-positive lipofilling sites was significantly shortened compared with that of the PRF-negative lipofilling sites.

**Conclusion:**

Facial filling with autologous fat grafts is effective and safe. Our results show that PRF does not markedly improve fat graft volume retention in the temple but significantly reduces postoperative recovery time.

**Trial Registration Number:**

ChiCTR2100053663.

## Introduction

Facial lipofilling surgery, which is a natural, long-lasting method of filling and supporting the face, has become increasingly popular. Absorption rates vary, probably due to variation in the techniques used (1). However, even with the same technique, some patients had unsatisfactory results after surgery in our department, and unabsorbed fat is a challenge for surgeons. Several techniques to improve volume retention have been described in the literature. The most interesting fat-grafting techniques use pro-survival strategies, such as platelet-rich plasma (PRP) (2–7) and platelet-rich fibrin (PRF) (8–10).

PRP is widely used in periodontal and oral surgery, spinal fusion, and cardiac bypass surgery, and successful clinical results have been achieved (11–14). Theoretically, PRP has the potential to enhance the survival of grafted fat. Although some studies demonstrate that the combination of fat grafts and PRP is successful (6, 15–18), others suggest that PRP is not effective (2, 19).

PRF was first described in France by Choukroun et al. in 2001 as a second-generation platelet concentrate technique (20, 21). PRF has several advantages over PRP, including a simpler production process, that makes it more suitable than PRP for widespread use in clinical settings. Nevertheless, objective evidence supporting the effectiveness of PRF in improving the retention volume of autologous fat grafts remains limited, and the current perceived benefits of PRF application in fat grafting are derived mostly by comparing the fat survival rate between PRF (added during lipofilling) and control groups (8, 22, 23). While previous studies provide valuable data on the evaluation of PRF, these are subjective and lack objective data. Thus, high-quality, prospective, randomized, controlled trials are imperative for the objective assessment of PRF efficacy in fat graft retention in clinical settings. This study aimed to evaluate the efficacy of PRF in facial lipofilling.

## Patients and Methods

### Demographic Characteristics

This controlled, split-face, randomized trial included a total of 20 female patients aged 28–50 years (mean age 38.6 ± 7.2 years) with a body mass index (BMI) of 18.1–23.0 kg/m^2^ (mean 20.3 ± 2.4) who received fat grafts for facial lipofilling (temple region) between January 2018 and May 2019 in our department. Patients who underwent unilateral facial lipofilling surgery; had other operations around the facial region; underwent additional procedures, such as a facelift or blepharoplasty; could not be followed up for 3 months; or whose postoperative BMI changed significantly were excluded from this study. Patients served as their own controls: the two temple regions of each patient were randomly designated as either the PRF side or the control side.

The study protocol complied with the Declaration of Helsinki and was approved by the Xijing Hospital Ethics Committee (No: LL-KY-20131226). Written informed consent was obtained from each patient.

### Fat Harvesting and Processing

All patients were examined preoperatively to estimate the total amount of fat to be transplanted into the temples. All patients received general anesthesia for the operation, and all of the steps involved in fat harvesting, processing, and injection were performed by the same surgeon according to the protocols described by Coleman (24).

A 3 mm incision was made with a scalpel blade at the thigh area, and an anesthetic solution containing 500 ml saline solution, 20 ml of 20 mg/ml lidocaine, and 0.5 ml of 1 mg/ml epinephrine was administered into the donor area through a blunt cannula. Subsequently, adipose tissue was harvested through the same incision with a two-hole, blunt Coleman harvesting cannula (2 mm in diameter, 15–23 cm in length) that was attached to a 10 ml Luer-Lok syringe whose plunger was pulled back to the 1 ml marker to maintain a gentle negative pressure. Once filled with lipoaspirate, the syringe was placed into a sterilized container and was spun in a centrifuge for 3 min at 3,000 rpm, which separated the lipoaspirate into three layers. The upper layer consisted primarily of oil and was decanted. The lower layer, which contained blood, water, and any aqueous elements, was discarded. The middle layer contained adipose tissues, which were collected for grafting.

### PRF Preparation

**Figure 1:**
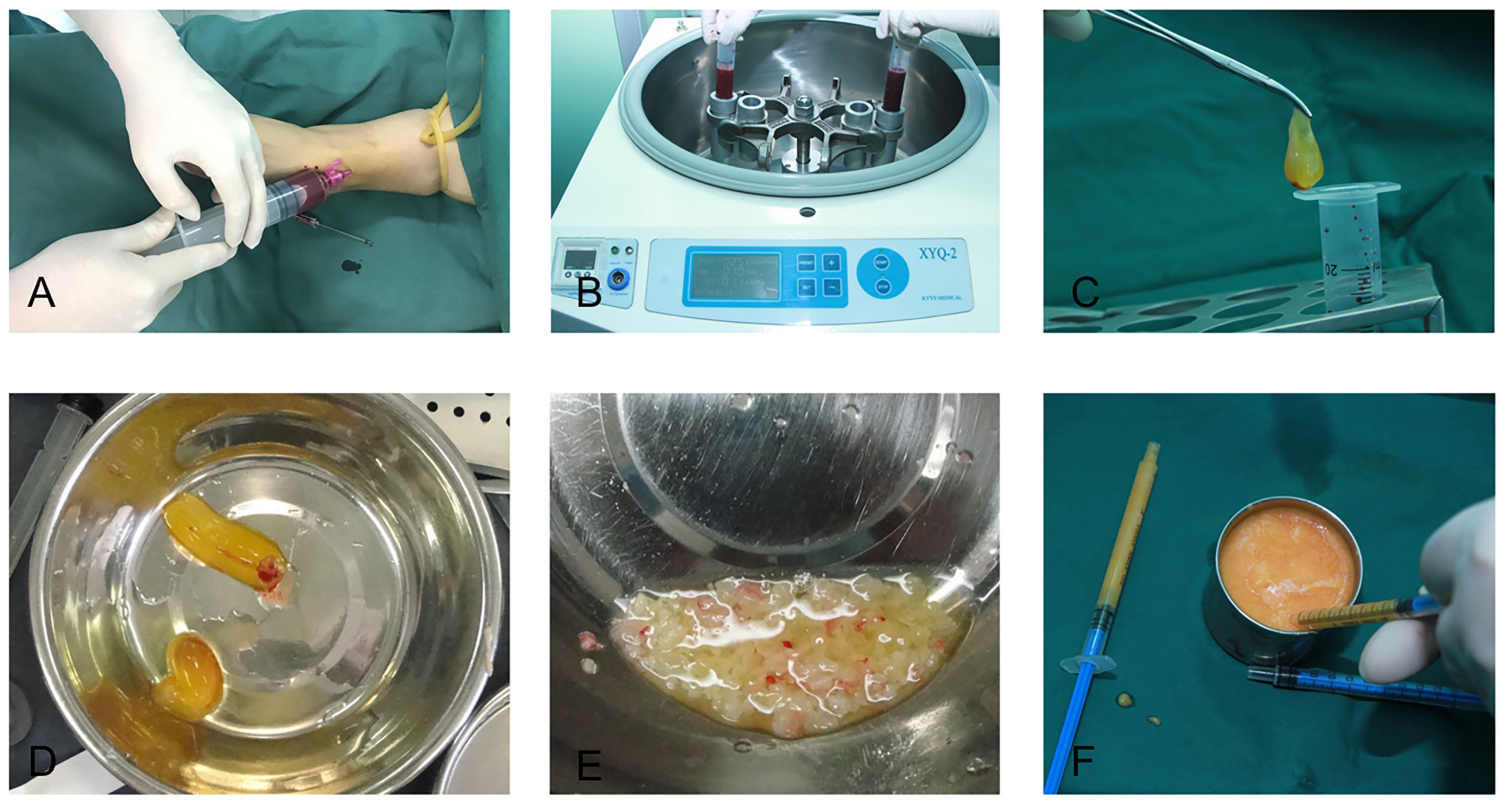
Platelet-rich fibrin preparation. **(A)** Intravenous blood was collected with a disposable syringe. **(B)** The blood was centrifuged at 3,000 rpm for 10 min. **(C)** A platelet-rich fibrin clot was taken out of the syringe. **(D)** A platelet-rich fibrin clot. **(E)** The platelet-rich fibrin clot was cut into fragments of 1 × 1 mm. **(F)** The fragments of the platelet-rich fibrin clot were mixed with the refined fat, and the mixture was subsequently transferred to 1 ml Luer-Lok syringes for injection.

Depending on the amount of fat that would be transplanted for each patient, 20–60 ml of intravenous blood was collected, immediately distributed into several disposable tubes containing no anticoagulant agents and centrifuged at 3,000 rpm for 10 min. The protocol for PRF preparation was as described by Choukroun et al. (20, 21). The speed of blood collection and transfer to the centrifuge was vital in obtaining a clinically usable PRF clot (8). After centrifugation, the PRF clot, which was located between the upper layer of acellular plasma and the bottom layer of red blood corpuscles, was harvested, placed in a sterile dish and cut into 1 × 1 mm fragments with microsurgical scissors. The fragments were then mixed with the centrifuged middle layer fat grafts at a volume ratio of 1:2. This mixture was thoroughly blended by repeated stirring with a pair of forceps before transferring it into several 1 ml Luer-Lok syringes for lipofilling in one temple (Figure 1); lipofilling for the other temple was performed using a mixture of saline, at the same volume as the PRF clot, and fat.

### Fat Injection

The anesthetic solution was bilaterally administered into the subcutaneous tissue of the temple using an 18-gauge needle, which passed through the skin via 2 mm incisions made along the hairline to prevent bruising and vascular cannulization. Incision sites were placed at frequent intervals. A 17-gauge blunt-tip cannula was attached to a fat-filled syringe and inserted into the subcutaneous layer of the temple until the cannula tip reached a required site. Thereafter, with the cannula being constantly withdrawn, fat grafts were injected into the appropriate plane. Each withdraw–inject action deposited ≤ 0.1 ml of fat graft inside the tunnel created by the cannula. Layers of fat were deposited by repeating injections, starting from the deepest layer in the recipient areas. The volume of fat injected volume is preliminarily judged by the volume of anesthetic agent injected, the volume was equal between fat and anesthetic agent. The incisions were closed with 7-0 nylon sutures after completion of the fat graft implantation procedure. After injection, the patients were required to sleep in supine position to avoid the fat out of shape.

### Effect Evaluation

**Figure 2:**
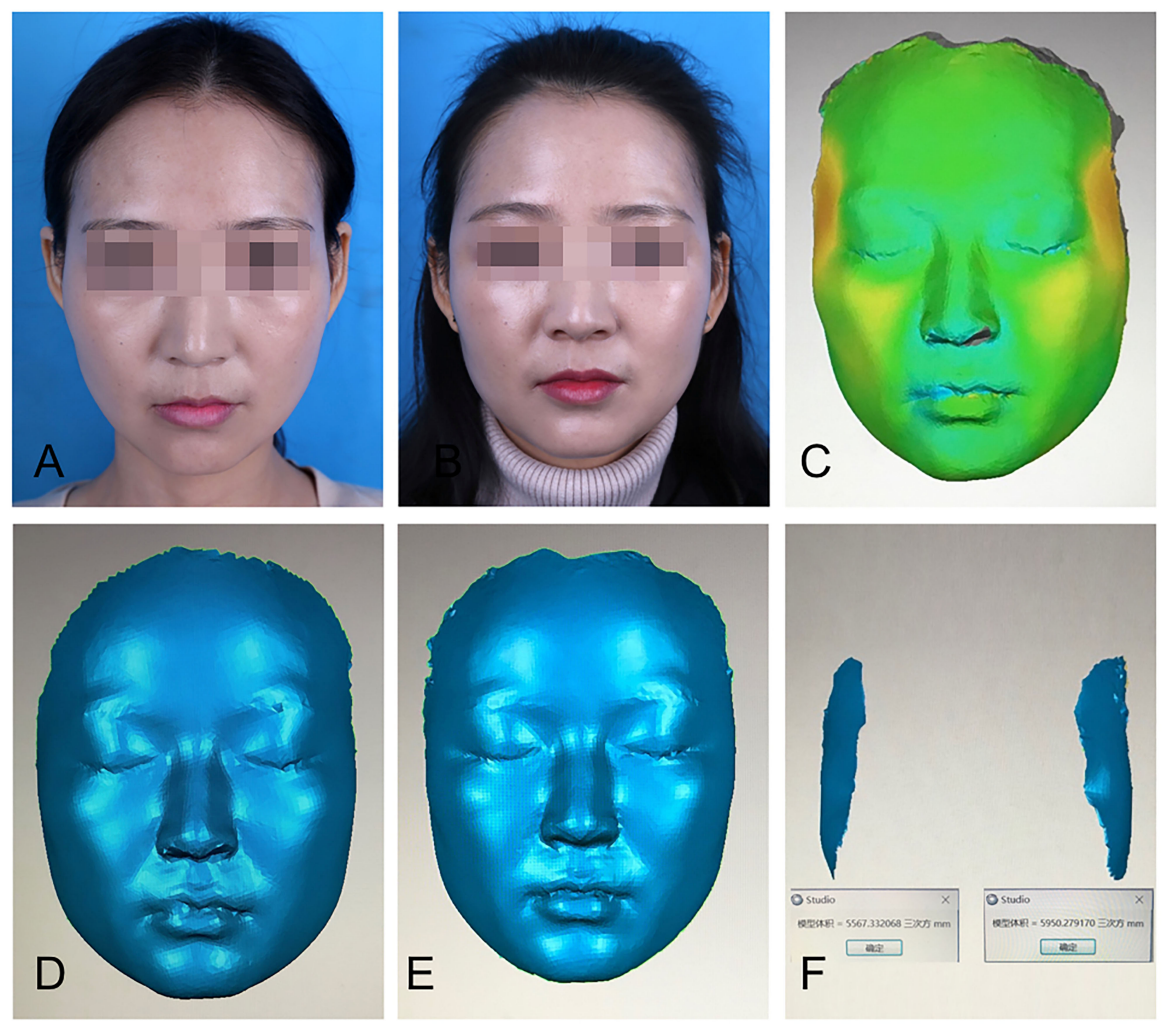
Three-dimensional (3D) reconstruction was used to calculate facial volume changes. **(A)** A patient's preoperative plain photograph. **(B)** Postoperative plain photograph 3 months after fat graft to the temples. **(C)** The pre- and postoperative images were automatically registered by Geomagic Studio 12 software, which produced 3D colorimetric topography for volumetric discrepancy analysis. **(D)** Preoperative 3D image. **(E)** Postoperative 3D image at 3 months. **(F)** 3D colorimetric analysis was used to calculate facial volume changes in each side.

Three approaches were employed to assess the effects of PRF on fat graft volume retention. The first one utilized three-dimensional (3D) photography technology, in which standardized photographs were obtained preoperatively and at 3 months postoperatively in three views using a 3D camera system. The pre- and postoperative scans were automatically registered by Geomagic Studio 12 software (Geomagic Solutions, Morrisville, NC). The data obtained were considered accurate and objective (Figure 2). In addition, the software produced 3D colorimetric topography, which was used to analyze the volumetric discrepancy in the temples before and after fat grafting. In the second approach, a questionnaire was completed by a plastic surgeon who did not perform the procedure and who was blinded to the assignment of fat grafting with or without PRF. The questionnaire was designed to evaluate the effects of fat graft implantation using a visual analog scale ranging from 1 to 5 (1 = no effect and 5 = significant effect). The scores provided by the surgeon were based on the evaluation of plain photographs of the patients, taken under the same conditions preoperatively and systematically at 3 months postoperatively. The last approach was a satisfaction assessment. Patients were asked to provide feedback through a questionnaire survey during the 3-month follow-up. The contents of the questionnaire included the following: overall patient satisfaction with the operation (scale ranging from 1 to 5), the number of days required before returning to work or resuming social activities without using camouflaging agents, and the incidence of complications.

### Statistical Analysis

Statistical analyses were performed with SPSS software (version 18.0; SPSS Inc., Chicago, IL, USA), and a paired-sample *t*-test was used. Data charts were generated using Prism 5.0 software (GraphPad Software, Inc., La Jolla, CA). Statistical significance was set at *p* ≤ 0.05.

## Results

**Figure 3:**
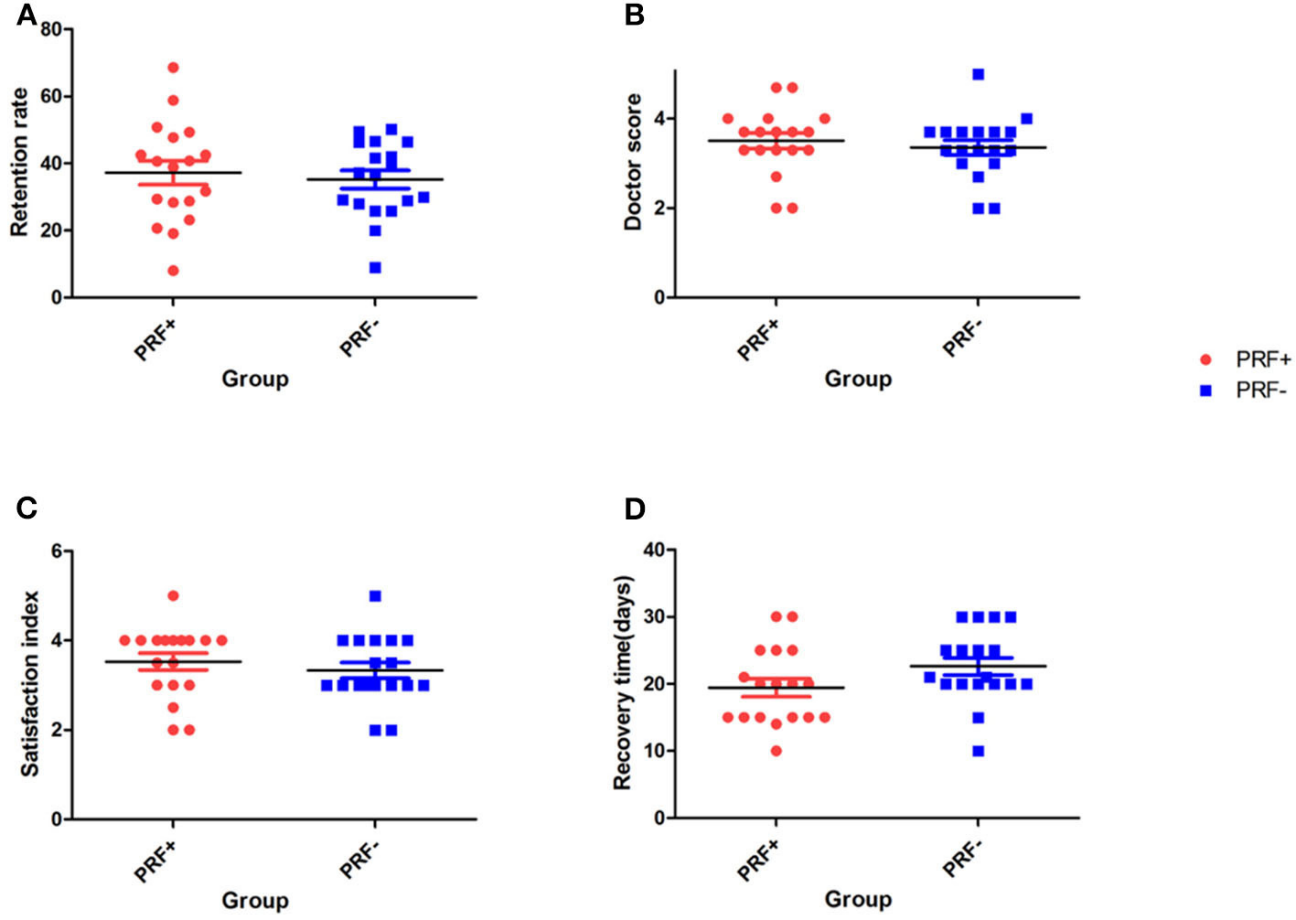
Scatter plot at 3 months. **(A)** The average fat retention rate at 3 months postoperatively. **(B)** Surgeon scores. **(C)** Patient satisfaction scores. **(D)** Patient recovery times.

Eighteen of the 20 enrolled patients completed the follow up project (two patients quit during the follow-up). The 3D-reconstruction analysis showed that the average fat retention rate at 3 months postoperatively was 37.21 ± 15.08% (mean ± SD) in the PRF-positive group and 35.20 ± 11.32% in the PRF-negative group. No statistically significant difference between the two groups was found (*p* = 0.37) (Table 1, Figure 3).

**Table 1:** Statistical analysis of retention rate between the two groups.

	**PRF group (*****n*** **= 18)**			**Control group (*****n*** **= 18)**			***P***
	**Mean**	**SD**	**Range**	**Mean**	**SD**	**Range**	
Volume of fat transfer	13.18	2.65	8–18	13.18	2.65	8–18	
Volume at 3 months after operation	4.16	2.21	0.97–10.98	3.87	1.58	1.08–7.92	
Fat retention rate (%)	37.21	15.08	8.04–68.64	35.20	11.32	8.96–50.74	0.37

*PRF, platelet-rich fibrin*.

*P-value was calculated by paired t-test*.

**Figure 4:**
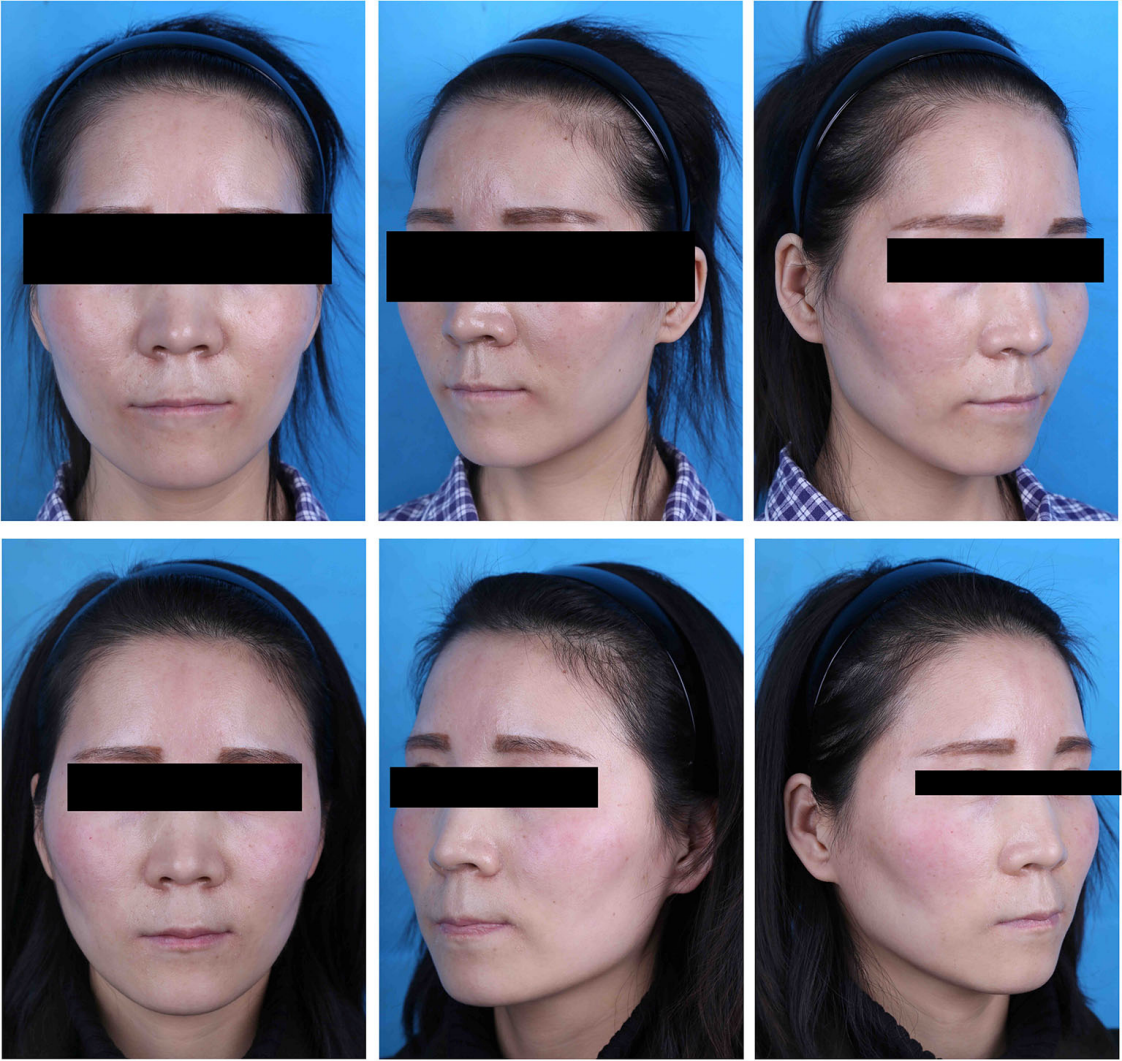
A case of a 28-year-old patient. (Top) Preoperative view. (Bottom) Postoperative view 3 months after fat graft to the temples; 16 ml of PRF-enhanced fat was transferred into the right temple, while the left side received the same volume of fat combined with saline.

The results of our follow-up questionnaires are summarized in Table 2 and Figure 3. Evaluation by the plastic surgeon revealed that the average score for the treatment outcome was 3.51 ± 0.73 in the PRF-positive group and 3.36 ± 0.69 in the PRF-negative group, and no significant difference between the groups was found (*p* = 0.17). The average patient satisfaction score in the PRF-positive group was 3.53 ± 0.79 (mean ± SD), whereas in the PRF-negative group, it was 3.33 ± 0.75. This difference was not significant (*p* = 0.33). The average recovery time was 19.44 ± 5.72 days in the PRF-positive group and 22.61 ± 5.45 days in the PRF-negative group, and a significant difference between the groups was noted (*p* = 0.02). An example of a typical case is shown in Figure 4.

**Table 2:** Results of surgeon's and patients' questionnaires.

	**PRF group (*****n*** **= 18)**			**Control group (*****n*** **= 18)**			***P***
	**Mean**	**SD**	**Range**	**Mean**	**SD**	**Range**	
Surgical outcome evaluated by an independent surgeon (1–5)	3.51	0.73	2–4.7	3.36	0.69	2–5	0.17
Overall patient satisfaction (1–5)	3.53	0.79	2–5	3.33	0.75	2–5	0.33
Recovery time (days)	19.44	5.72	14–30	22.61	5.45	10–30	0.02

*PRF, platelet-rich fibrin*.

*P-value was calculated by paired t-test*.

In this study, fat embolism, vascular/nerve injury, infection, necrosis, massive edema, prolonged bruising, or severe pain were not observed. Five patients in the PRF-positive group and eight in the PRF-negative group had postoperative hematoma, and all of these patients recovered after 2 weeks. Under-correction was the most frequent complication in both groups.

## Discussion

Autologous fat grafting, which was first reported in 1893 by Neuber (25), is now one of the most commonly performed procedures in plastic surgery. An increasing number of surgeons have considered fat tissue as the ideal facial filler mainly because it is readily available, safe, inexpensive, and easy to harvest (26). In this study, autologous fat grafting was used; thus, host rejection was avoided. As a facial filler, autologous fat tissue is soft and natural and may improve the skin's quality at no additional cost to patients (27). Nevertheless, the absorption rate of the grafted fat is high and difficult to predict (28). Although numerous efforts have been made to refine the grafting protocols, there has been no consensus among practitioners on a standardized method that improves the survival rate of fat grafts (29).

The Coleman protocol is currently the most widely adopted method for fat harvesting, processing, and injection. The clinical outcome has been confirmed by a large number of studies (30). However, even with the use of this method, the postoperative fat absorption rate remains high. To improve the survival rate of fat grafts, several strategies have been proposed, such as tissue engineering and the use of cell-stimulating factors (31–34). The implantation of autologous fat grafts mixed with PRP or PRF is another interesting strategy for improving graft survival rates.

PRP may stimulate the regeneration and repair of different tissues via the activation and substantial secretion of growth factors and cytokines stored in cellular alpha-granules. These may affect cell proliferation, chemotaxis, angiogenesis, cell differentiation, fibro-genetic activity of fibroblasts, and synthesis of the extracellular matrix (35). PRP is widely used in periodontal and oral surgery, spinal fusion, and cardiac bypass surgery, and successful clinical results have been achieved (11–14). Theoretically, PRP has the potential to enhance the survival of grafted fat, and the effects of the combination of fat grafts and PRP have been evaluated in some animal experiments and clinical trials. Although some of the experimental results proved the effectiveness of PRP (6, 15–18, 36, 37), others demonstrated no significant improvement in the fat graft necrosis rate (2, 19). One of the main limitations of PRP is that most of the pre-synthesized growth factors are secreted within the first hour after mixing and grafting with fat. Only a few growth factors show additional synthesis up until day 8 (38). This means that fewer growth factors play a significant role in fat graft survival. Furthermore, PRP preparation is complex, and anticoagulants are needed during the process.

In 2001, PRF, a second-generation platelet concentrate technique, was first described in France by Choukroun et al. (20, 21). It has several advantages over PRP, such as ease of preparation and absence of a biochemical modification requirement. In contrast to PRP, several studies have confirmed that PRF gradually releases the platelet-derived growth factor and the transforming growth factor for 28 days *in vitro* (9, 39). Moreover, a study by Dohan et al. proved that the leucocytes and platelets trapped in the fibrin matrix continue to produce moderate quantities of cytokines, which may intervene in the regulation of inflammatory reactions, thereby leading to enhanced protection from infection (40). Unlike PRP, PRF does not dissolve quickly after application. The strong fibrin matrix is slowly remodeled in a way similar to that in a natural blood clot. The platelets and released growth factors are combined in a chemical bond. As they are released slowly, activity of the growth factors is prolonged, which improves fat graft survival (10). Xiong et al. concluded that PRF increased tissue retention, quality, and vascularization of grafted fat compared with PRP rabbits (41). Thus, PRF seems to be more suitable than PRP for widespread use in clinical settings.

Furthermore, there have been several clinical observations of decreased absorption of fat grafts after facial lipofilling using mixtures of PRF and autologous fat, which could be associated with the actions of PRF (8, 22). However, data from high-quality, prospective, randomized, controlled trials are still needed to objectively evaluate PRF efficacy. Thus, we performed a controlled, split-face, randomized trial to investigate the possible beneficial effects of adding PRF to aesthetic facial lipofilling in a well-defined healthy patient cohort. We used 3D photographic reconstruction to detect facial fat graft retention, which was first reported by Meier et al. (42), for our evaluation to provide accurate and objective data. The results demonstrated that the addition of PRF to the fat graft did not significantly increase the fat survival rate, although other benefits such as reduction of patients' postoperative recovery time and enhanced overall patient satisfaction were observed. Unlike PRP (43–45), PRF has never been reported to be associated with a reduction in patient recovery time after lipofilling.

The PRP or PRF dose should be considered when evaluating any possible effect on fat graft survival. Based on a previous investigation, PRP may have a dose-dependent positive effect on fat grafts, and the concentration of PRP that appears to achieve the most favorable results ranges from 0.4 to 0.5 ml per ml of fat (1:2 ratio) (46). Other studies show that a higher concentration of PRP may induce unwanted cell differentiation resulting in counterproductive effects (47, 48). However, the mixing ratio of PRF to fat was not established yet. Yu et al. [49] concluded that the best promoting effect when the mixing ratio of PRF to fat is 1:10. However, the study was conducted in mice. In our study, the selected ratio of PRF to fat was 1:2, which was identical to that used in PRP studies. However, the effects of the PRF dose were not evaluated in this study. Thus, further investigation is required to clarify the effect of PRF on fat graft survival.

This study has some limitations that need discussion. First, the patients were only followed up for 3 months postoperatively, and the sample size was small. Longer follow up will make the results more convincing. Second, the mixed ratio of PRF to fat was 1:2, and the ratio was not widely acknowledged. Further studies are needed to determine the optimal mixed ratio. Third, the equal injection method was used in our study. The under-correction was the most frequent complication in our study due to the fat absorption after operation. If overcorrection method was used, PRF may play a better role in fat survival, and the results may also change accordingly. However, further study is necessary to identify the assumption.

In conclusion, autologous fat grafting remains a good choice in facial aesthetic surgery because it is a safe, effective, and durable treatment. Our study has shown that PRF does not improve fat graft volume retention in the temples. Nevertheless, it could significantly reduce postoperative recovery time. The use of platelet concentrates as an additive in lipofilling has been increasingly reported. However, the reproducibility of the reported positive effects has not been widely confirmed in randomized, controlled trials. Therefore, further research is warranted.

## Data Availability Statement

The original contributions presented in the study are included in the article/supplementary material, further inquiries can be directed to the corresponding author/s.

## Ethics Statement

The studies involving human participants were reviewed and approved by Xijing Hospital Ethics Committee (No: LL-KY-20131226). The patients/participants provided their written informed consent to participate in this study. Written informed consent was obtained from the individual(s) for the publication of any potentially identifiable images or data included in this article.

## Author Contributions

Z-XZ and L-HQ was involved in drafting the manuscript and revising it critically for important intellectual content and they made substantial contributions to conception and design of the work. NS, S-HX, and X-JM made substantial contributions to the acquisition, analysis, and interpretation of data for the work. C-GY participated in supervision and data interpretation. All authors read and approved the final manuscript.

## Funding

This study was supported by the National Natural Science Foundation of China (81671932 and 81701919).

## Conflict of Interest

The authors declare that the research was conducted in the absence of any commercial or financial relationships that could be construed as a potential conflict of interest.

## Publisher's Note

All claims expressed in this article are solely those of the authors and do not necessarily represent those of their affiliated organizations, or those of the publisher, the editors and the reviewers. Any product that may be evaluated in this article, or claim that may be made by its manufacturer, is not guaranteed or endorsed by the publisher.
